# Telehealth in South Africa: A guide for healthcare practitioners in primary care

**DOI:** 10.4102/safp.v64i1.5533

**Published:** 2022-06-28

**Authors:** Mareike Rabe

**Affiliations:** 1 Family Physician, Locum in Public and Private Practice, Cape Town, South Africa

**Keywords:** telehealth, virtual consultations, remote consultations, primary care, guidelines, ethics, medicolegal, communication

## Abstract

The use of telehealth is becoming a prevalent feature in clinical practice worldwide, partly because of advances in medical and telecommunications technology. The coronavirus disease 2019 (COVID-19) pandemic has been a key driver in justifying the accelerated use of telehealth, leading to healthcare practitioners (HCPs) utilising virtual consultations more avidly. Although challenges remain, recent data have shown that remote consultations are feasible, safe and effective in South Africa (SA) and that HCPs should become proficient in conducting telehealth, virtual or remote consultations. These guidelines are based on the revised Health Professions Council of South Africa (HPCSA) General Ethical Guidelines for Good Practice in Telehealth (Booklet 10) and guidelines on remote or video consultations from the University of Oxford, the Royal Australian College of General Practitioners and the Royal College of Psychiatrists. These guidelines aim to equip HCPs with the basic knowledge and skills pertaining to medicolegal, communication and practical aspects of telehealth and how to practise telehealth safely and effectively in primary care settings in SA during the COVID-19 pandemic and beyond.

## Introduction

The ongoing coronavirus disease 2019 (COVID-19) pandemic has forced healthcare practitioners (HCPs) to reconsider and reinvent their interactions with patients. Social distancing and the redeployment of HCPs to fight the pandemic meant that conventional physical or face-to-face consultations were no longer feasible, acceptable and accessible to many patients and HCPs alike. The term ‘telehealth’ is preferred over the previously used term ‘telemedicine’ as it is more inclusive and accommodates all relevant professionals who practise telehealth.^1^ Telehealth has been practised by HCPs for many years, especially by telephone.^2^ Video consultations have become a more prominent feature of telehealth service delivery globally, including in sub-Saharan Africa (SSA), although many challenges remain regardless of the platform used. These challenges include infrastructural, technological, organisational, legal and regulatory, ethical, knowledge and awareness, financial and cultural resistance barriers to telehealth implementation.^3,4,5,6^

Promisingly, a study performed during the first wave of the COVID-19 pandemic in the Western Cape province has shown that a dedicated telehealth service led to positive outcomes for specific patient groups and HCPs and demonstrated the development of virtual doctor–patient relationships, a decrease in mortality and an increase in cost-effectiveness.^7^ Healthcare practitioners should be mindful that the aforementioned study was conducted within the context of the COVID-19 pandemic and further research is needed to assess the performance of a dedicated telehealth service in the context of general healthcare consultations.

A systematic review by Mbunge et al. compiled five recommendations for the effective integration of telehealth service in SSA. Firstly, international organisations such as the World Health Organization (WHO) should lead the way by conducting training workshops with HCPs on the potential role of telehealth in providing quality healthcare in SSA. Secondly, standard telehealth policies and frameworks should be designed specifically for the SSA context to address the needs of HCPs who practise telehealth in the region. Thirdly, import duties on telemedicine equipment should be done away with or subsidised by governments to encourage the use of telehealth hardware and software amongst HCPs. Fourthly, multisectoral consultative processes which involve community members should be undertaken to create and expand networks and bridge the current digital divide in SSA. Finally, regulatory procedures around telehealth service delivery should be provided by regulatory authorities in a robust and unrestricted manner.^5^

Heeding the lessons learned during COVID-19 pandemic, HCPs in South Africa (SA) should become well-equipped to deliver healthcare services sufficiently in this new era of telehealth. Its application is specifically useful in certain population groups such as people who are incarcerated, people who live with disabilities, people who require psychotherapy or physical therapy and patients with well-controlled chronic diseases who require prescription renewals.^8^

Telehealth is defined as the application of electronic telecommunications, information technology or other electronic means to administer healthcare services in two separate geographic locations for the purpose of facilitating, improving and enhancing clinical, educational and research outcomes, particularly to under-serviced communities in SA. Telehealth is a blanket term that covers all components and activities of healthcare and the healthcare system that are conducted through telecommunications technology.^1^ Telemedicine services on the other hand include teleconsultation, telecounselling, telescreening, telediagnosis, real-time communication and telemonitoring.^5^ Telehealth services may be delivered synchronously or asynchronously. The former refers to telehealth services that are delivered in real time, such as by telephone consultations. The latter refers to telehealth services that are not delivered in real time, for example, using email to communicate.^1^

According to a general household survey conducted by Statistics SA in 2017, 16.9% of South Africans (or 9.5 million people) were covered by a medical aid scheme.^9^ This means that at least 45 million South Africans are still largely dependent on public healthcare.^10^ Over 36 million South Africans accessed the Internet through any kind of mobile device in 2021. This figure is projected to increase by 17.5% in 2026, meaning that 43 million South Africans will be mobile Internet users.^11^ One-third or about 22 million people in SA currently use smartphones.^12^ Based on the above information, these guidelines are aimed at HCPs who work in the public health sector of SA, as this group constitutes the majority of the South African population.

These guidelines are aligned and should be read in conjunction with the revised Health Professions Council of South Africa (HPCSA) General Ethical Guidelines for Good Practice in Telehealth (Booklet 10, the term which will be used to refer to these guidelines throughout this article)^1^ as well as published guidelines about telehealth, remote and video consulting from the University of Oxford, the Royal Australian College of General Practitioners (RACGP) and the Royal College of Psychiatrists (RCP).^13,14,15,16^

These guidelines aim to equip HCPs with the basic knowledge and skills pertaining to medicolegal, communication and practical aspects of telehealth and how to practise telehealth safely and effectively in primary care settings in SA during the COVID-19 pandemic and beyond. These guidelines should be seen as an introductory overview of telehealth and its application in the context of low-income and middle-income countries in SSA where telephone consultations may be more feasible, with the view of expanding to video consultations as resources and technology become increasingly available and accessible.

## How to navigate the telehealth medicolegal landscape in South Africa

### Should telehealth consultations be viewed in the same way as physical consultations by healthcare practitioners?

Healthcare practitioners who conduct telehealth consultations *are held to the same standards of professional practice* as HCPs who conduct physical consultations and will be measured against what the reasonable HCP would do under similar circumstances should complaints arise or legal action ensue in this milieu.^1^ Do not supersede or go above and beyond what you would do during a physical consultation when conducting a telehealth consultation. For example, during a telephone consultation, do not diagnose and/or treat atrial fibrillation in a patient who was not known to have this condition previously and has not undergone a physical examination and electrocardiogram (ECG) analysis to confirm the arrhythmia. Other questions HCPs should ask include:^13^

- How do you normally prepare for or conduct a physical consultation?
- How would you shift these preparations for a telephone or video consultation?
- What would you need to change to ensure the same level of assessment and care is conducted if you are using a telephone or video consultation?

In both the public and private health sectors, it is pertinent to develop standard operating procedures (SOPs) and protocols pertaining to telehealth services. Quality improvement principles could be implemented to ensure the appropriate allocation of resources.^17^ The steps could include an initial assessment of patients’ views around the feasibility and acceptability of telephone or video consultations in their facility or setting. Flowing from these inputs, online appointment schedules for outpatient department visits could be set up by using a booking system that works for the facility. Healthcare practitioners could then be allocated to ‘virtual queues’ to screen and triage patients seeking chronic care and those who have undifferentiated or acute problems. It is imperative that a safeguard system is in place to ensure that, should patients need to undergo a physical examination or further care outside the scope of what telehealth can provide, they know where to go and how to access these services in a timely manner.

### Should a relationship exist between healthcare practitioners and patients before telehealth consultations occur?

Prior to the revision of Booklet 10 in December 2021, the HPCSA advised that telehealth consultations should occur between HCPs and patients only when they had established professional relationships. This meant that the patient’s health records had to be available to the treating HCP, and/or the patient was known to the HCP prior to the telehealth interaction between the two parties.^18^ These guidelines were applicable in a context where a ‘servicing’ HCP who consulted with the patient face-to-face treated the patient in conjunction with a ‘consulting’ HCP after attaining the latter’s input from a remote location. For example, a community service doctor (the servicing HCP in a primary care clinic) shares relevant clinical, laboratory and radiological information about a patient who underwent a tuberculosis work-up with a senior colleague (the consulting HCP in a district hospital) to decide on appropriate further management of the patient. The revised guidelines state that although an established professional relationship is *desirable*, it is not a compulsory prerequisite for telehealth interactions between HCPs and patients. When a HCP agrees to treat a patient and the latter provides informed consent to be treated, the professional relationship is established according to the updated Booklet 10.^1^

Booklet 10 further states that telehealth consultations are *not* a substitute for physical consultations but should resemble them as closely as possible. Healthcare practitioners should *not* exclusively use telehealth consultations to deliver healthcare services in their practice; that is, physical consultations should still be the foundation of healthcare service delivery. Telehealth interactions should only be used when it is in the best clinical interest of patients.^1^

### Which device(s) and/or platform(s) should be used to conduct telehealth consultations?

Booklet 10 clearly states that ‘any suitable Information and Communication Technology (ICT) platforms’ may be used to ‘exchange information for the diagnosis and treatment of diseases and injuries, research, and evaluation, and for the continuing education of HCPs’.^1^ Booklet 10, however, discourages the use of social media platforms to communicate with patients as ‘a failure to maintain strictly professional relationships with patients could result in ethical dilemmas’.^1^ The onus rests on HCPs to ensure that the device(s) and platform(s) used to deliver telehealth services are password-protected with updated antivirus and other software, such as virtual private networks (VPNs), to ensure patient confidentiality is maintained and interactions are secured and aligned with the Protection of Personal Information Act (POPIA).^19^

### When is it unsafe to conduct a telehealth consultation?

The decision to offer a remote consultation as opposed to a physical consultation should always take into account the following:^13,14,15,16^

- The potential for serious, high-risk conditions where a physical examination is necessary – for example, in a patient known with ischaemic heart disease who complains of more frequently occurring chest pain, chest pain that lasts for longer periods of time, chest pain at rest, or chest pain which is not relieved by standard therapy.
- Where a physical examination cannot be deferred or is not possible to conduct virtually, such as an internal gynaecological examination.
- Comorbidities or conditions which may affect the patient’s ability to use the technology – for example, advanced dementia or serious anxiety about or refusal to use the technology.
- Patient vulnerability – for example, mental healthcare users, children, people who live with disabilities (including people who are sight- and/or hearing-impaired), older age, multimorbidity.

Should HCPs have *any* uncertainty that a telehealth consultation is not in the best interest of the patient, a physical consultation should be recommended and arranged. On the other hand, HCPs should guard against bias and generalisation towards certain population groups in terms of the suitability of a telehealth consultation and consider each case individually.^16^ For example, an older person may be well-equipped and knowledgeable about the use of technology for video interactions, but the HCP assumes that this is not suitable without checking with the patient. Similarly, it is important to consider current epidemiological factors such as peaks of disease outbreaks and risk stratification for high-risk individuals, such as during the COVID-19 pandemic.^16^

### How should record-keeping occur for telehealth consultations?

As is the case when conducting physical consultations, record-keeping during telehealth interactions is *vitally* important. Clinical notes should be handwritten or captured electronically and stored in a manner to maintain patient privacy and confidentiality. Healthcare practitioners should be mindful to not appear distracted by writing extensive clinical notes during telehealth consultations and allow ample time between consultations to ensure that patient records are updated. The Medical Protection Society (MPS) recommends that telehealth consultations should not be recorded by HCPs. It should be standard practice for HCPs to inform patients that telehealth consultations are live-streamed and check with patients whether they are recording interactions. If a patient reports that they are recording the interaction, their reasons should be explored respectfully and documented. Healthcare practitioners cannot force patients to stop recording interactions or refuse to continue with interactions when being recorded. If a physical consultation is deemed necessary in such circumstances, consultations should be converted appropriately.^20^

## A proposed template for initiating a telehealth consultation

‘Hello, my name is Doctor …

Before we continue, may I confirm that you can see and hear me clearly?

Thank you for completing the triage and consent questionnaires earlier. May I check whether anything has changed since you completed these questionnaires?

May I check that you are Mrs …, residing at … and that your date of birth is …?

Are you currently at your place of residence or elsewhere?

This consultation is being live-streamed, which means that I am not recording this interaction. May I check whether you are recording this interaction?

There is no one else in the room with me; may I check whether someone is with you at the moment? May we speak freely?

If there is a technical or other problem during this consultation, may I confirm that the alternative number where I may reach you is …?

I will be making notes now and then to make sure I don’t miss anything, but you will still have my full attention even if I don’t look at you all the time.

What were you hoping I would do for you today?’

Table 1 summarises important information which should be documented for telehealth interactions. It also provides a practical how-to approach to telehealth consultations. Table 1 is adapted from resources of the University of Oxford and the RACGP, as well as the RCP, specifically relating to the ‘6 Cs’ of video consultation (competence, confidence, consent, confidentiality, communication and contingencies), which are integrated throughout the table.^13,14,15,16^

**TABLE 1:** How to interact with patients remotely: communication considerations and practicalities.

Aspects of the consultation	Objectives	Prerequisites and examples
**Preparation** Including practice and workflow setup, triage and consent	To ensure a seamless consultation between HCPs and patients by developing *competence* in using the intended device or platform	*Practice setup*: Staff training, trial runs, suitable hardware and software, uninterrupted electricity supply, well-lit and private consulting rooms, a fast broadband connection.
		*Workflow setup*: Update practice website with information on consultation schedule, put processes in place for scheduled and unscheduled appointments, put arrangements in place for in-person contact (such as collection of forms for further investigation), ensure e-prescribing and e-referral pathways are set up.
		*Before the consultation*: ensure technology works on both ends; appear professional; avoid interruptions; familiarise yourself with clinical records, results and reports; set agenda to check and align with the patient’s agenda once interaction commences.
	To ascertain whether it is safe to continue with a consultation or whether the patient requires a physical consultation or should seek urgent or emergent care	Pre-populated triage and consent questionnaires could be completed by the patient at the time of booking the appointment or before the consultation commences. Examples and templates of such forms can be found at: https://www.jotform.com/form-templates/category/health and can be modified to fit the practice and patient profile.
		Every practice or department should have a protocol in place to direct patients to appropriate facilities for physical consultation, urgent or emergent care if indicated.
	To ensure the patient understands the limitations, risks and benefits of a telehealth consultation by providing informed *consent*	Although consent is *implied* by the patient if a consultation is attended remotely, consent should be gained *explicitly* and not assumed. It should be made clear during the consent process that, should the HCP feel at any point during the consultation that a physical examination is required, the patient should be directed to an appropriate facility where a physical examination can be done.
**Initiating the consultation** Including the date, time and place of the consultation, virtual platform on which the consultation is performed, the absence or presence of a chaperone and an appropriate greeting	To ensure the correct patient is attending the consultation and to create a comfortable environment (‘break the ice’) for the patient, especially if it is a new or unknown patient	First impressions set the scene for the rest of the consultation. ‘Webside manner’ is established by eye contact, facial expression, speech pattern, gesturing, positioning, lighting, background and showing *confidence* in using the device and platform. Check patient details with at least two identifiers – for example, patient’s name, residential address and date of birth.
		Initiate the consultation by an opening sentence such as ‘It has taken me a while to get used to “the new normal.” What has your experience been?’
	To ascertain whether to continue with or end the consultation and convert to a physical consultation or refer the patient appropriately	Does the patient look or sound acutely ill?
		For patients who are abroad, consultations should be discontinued after instituting safety measures – for example, advising patients to seek emergent care if indicated. Healthcare practitioners based in SA and registered with the HPCSA are not advised to deliver healthcare services to patients outside the borders of SA.^20^
	To gain an understanding of the patient’s current social circumstances	Patients may engage in consultations from a location other than their place of residence – for example, a family member’s house – if they are not comfortable to use the technology and need help to set up for the consultation.
	To align and rectify technological challenges on either side of the consultation	If the patient is struggling to connect on a video platform and it is established that a telephone consultation would suffice to reach the consultation’s objectives, this should be noted and a telephone consultation could proceed.
	To ensure that patient privacy and *confidentiality* are maintained	Caregivers, family members or interpreters may attend telehealth consultations with patients. Reasons could include that the patient is not able to provide informed consent or able to understand the treatment plan fully - for example, a language barrier exists. In these cases, the threshold to convert to a physical consultation should be low.
**History-taking and communication** Including the exploration of relevant positive and negative physical signs	To formulate a working diagnosis as accurately as possible with the information at hand whilst practising patient-centred *communication*	Ask open-ended questions, avoid medical jargon, explore the patient’s ideas, concerns and expectations, avoid interruptions and do not invalidate patients’ feelings.^21^
		Home test results such as blood pressure, temperature or glucose could be shared with the HCP.
		The clinical notes may indicate, for example, ‘Mr X reports experiencing diarrhoea with loose stools and stomach cramps for the past two days. The stools do not contain blood or mucous. He does not experience nausea, vomiting or abdominal distention and is able to tolerate fluids and solids. He has not taken any medication to alleviate the symptoms. No symptoms or signs of severe dehydration were elicited as per telephone consultation.’
**Summarising and feedback**	To formulate a working diagnosis to the highest degree of certainty and an appropriate management plan in collaboration with the patient	Always exclude and document danger signs and red flags and inform the patient when to seek further care. The clinical reasoning of how the HCP reaches diagnoses and provides management plans should be shared with patients. Encourage patients to write down important information or questions. The tell-back collaborative inquiry^22^ could be used to check understanding – for example, ‘I imagine you’re really worried about this rash. I’ve given you a lot of information. It would be helpful to me to hear what you will tell your husband regarding what we discussed about this rash and its treatment.’
**Plans, advice and action**	To negotiate the most appropriate, evidence-based and available treatment options with the patient	Explore the patient’s expectations and ideally use shared decision-making to decide on the way forward; for example, ‘You mentioned that you think you need an X-ray to assess the headaches you’ve been experiencing recently. Let’s talk about why you think that would change our management of the problem’.
		Non-pharmacological interventions and prescriptions should be shared electronically and referral to appropriate allied health professionals or specialists initiated if indicated.
**Safety-netting, closing the consultation and follow-up**	To have *contingencies* in place in the event that a patient cannot be contacted	Have a protocol in place should patients fail to attend appointments or if technology fails, especially for high-risk vulnerable patients such as mental healthcare users. Clearly document how many attempts were made to contact the patient and how this was done - for example, ‘Ms. Z did not attend her virtual consultation as per the appointment system. I phoned her on the provided home telephone and cellphone numbers for a total of four attempts between 11:00 and 12:00. All attempts were unsuccessful. I asked the receptionist to contact her by telephone and via email to see whether the appointment should be rescheduled or canceled.’ An emergency contact number should be available. Patients should clearly state what information may be shared via alternative means of communication; for example, patients could agree to be reminded of an appointment per email, but blood results should not be shared via this email address as it is a shared email address.
	To ensure continuity of care and appropriate further care should first-line treatment outcomes be suboptimal, treatment ineffective or patients deteriorate despite appropriate treatment	Patients should know what to look for, when to act, what to do, where to go and who to call if they don’t improve or deteriorate clinically.^20^ For example, ‘We have discussed the way forward in treating your allergic rhinitis. Should you not improve despite taking the treatment correctly in the next two or three days, please make an appointment to see me in person so I may examine you to decide whether further treatment or referral is necessary. Also contact me should you develop a fever, a frontal headache, a purulent nasal discharge or tenderness or pain over one or more sinuses.’
		Sign-post that you will sign off, wish the patient better and greet the patient. Ensure enough time for record-keeping activities before the next consultation commences.
		Allow feedback from patients about their experience via anonymous questionnaires or other tools.

*Source*: Please see the full reference list of the article, Video consultations: A guide for practice – Br J Gen Pract Life [homepage on the Internet]. 2020 [cited 2022 Mar 15]. Available from: https://bjgplife.com/video-consultations-guide-for-practice/, for more information

Note: ‘Consultation’ refers to virtual, remote or telehealth interaction.

HCP, healthcare practitioner; HPCSA, Health Professions Council of South Africa; SA, South Africa.

## Conclusion

The manner in which HCPs engage with patients and vice versa has changed considerably during recent years, partly because of medical and telecommunications advances. The COVID-19 pandemic has caused an escalation in the use of telehealth in everyday medical practice. Healthcare practitioners should become proficient in terms of basic knowledge and skills to navigate this new landscape so as to ensure patients are assisted in an ethical, systematic, efficient and safe manner using different platforms.

### Further reading

These guidelines provide an introductory overview to telehealth in SA. It is beyond the scope of this article to address subjects such as how to conduct remote consultations in specific specialities of clinical practice, how to interact with colleagues remotely (between the so-called servicing and consulting HCPs), how to break bad news remotely, how to e-prescribe and how to use social media in clinical practice. The author recommends the following resources for further reading:

- HPCSA Booklets 1 (General ethical guidelines for health care professions), 4 (Seeking patients’ informed consent: The ethical considerations), 5 (Confidentiality: Protecting and providing information), 9 (Guidelines on Patient Records) and 16 (Ethical Guidelines on Social Media); available from: https://www.hpcsa.co.za/?contentId=79.
- Implementation of electronic scripts in South Africa. Available from: http://www.samj.org.za/index.php/samj/article/view/9374
- MPS Remote Consulting Course and Webinar series. Available from: https://prism.medicalprotection.org/ (available to MPS members)
- Patient-Centred Communication: Basic Skills. Available from: https://www.aafp.org/afp/2017/0101/p29.html
